# Habit Strength, Medication Adherence, and Habit-Based Mobile Health Interventions Across Chronic Medical Conditions: Systematic Review

**DOI:** 10.2196/17883

**Published:** 2020-04-28

**Authors:** Sherif M Badawy, Richa Shah, Usman Beg, Mallorie B Heneghan

**Affiliations:** 1 Department of Pediatrics Northwestern University Feinberg School of Medicine Chicago, IL United States; 2 Division of Hematology Oncology and Stem Cell Transplant Ann & Robert H. Lurie Children's Hospital of Chicago Chicago, IL United States; 3 Weinberg College of Arts and Sciences Northwestern University Evanston, IL United States; 4 Midwestern University Arizona College of Osteopathic Medicine Glendale, AZ United States

**Keywords:** habit strength, medication adherence, habit index, medication compliance, mobile health, health, digital health, interventions, mobile phone

## Abstract

**Background:**

Unintentional medication nonadherence is common and has been associated with poor health outcomes and increased health care costs. Earlier research demonstrated a relationship between habit strength and medication adherence. Previous research also examined a habit’s direct effect on adherence and how habit interacts with more conscious factors to influence or overrule them. However, the relationship between habit and adherence and the role of habit-based mobile health (mHealth) interventions remain unclear.

**Objective:**

This review aimed to systematically evaluate the most recent evidence for habit strength, medication adherence, and habit-based mHealth interventions across chronic medical conditions.

**Methods:**

A keyword search with combinations of the terms *habit*, *habit strength*, *habit index*, *medication adherence*, and *medication compliance* was conducted on the PubMed database. After duplicates were removed, two authors conducted independent abstract and full-text screening. The guidelines for the Preferred Reporting Items for Systematic Reviews and Meta-Analyses (PRISMA) were followed when reporting evidence across the included and reviewed studies.

**Results:**

Of the 687 records examined, 11 met the predefined inclusion criteria and were finalized for data extraction, grading, and synthesis. Most included studies (6/11, 55%) were cross-sectional and used a theoretical model (8/11, 73%). The majority of studies measured habit strength using the self-report habit index and self-report behavioral automaticity index (9/11, 82%). Habit strength was positively correlated with medication adherence in most studies (10/11, 91%). Habit mediated the effects of self-efficacy on medication adherence (1/11, 9%), and social norms moderated the effects of habit strength on medication adherence (1/11, 9%). Habit strength also moderated the effects of poor mental health symptoms and medication adherence (1/11, 9%). None of the included studies reported on using or proposing a habit-based mHealth behavioral intervention to promote medication adherence.

**Conclusions:**

Habit strength was strongly correlated with medication adherence, and stronger habit was associated with higher medication adherence rates, regardless of the theoretical model and/or guiding framework. Habit-based interventions should be used to increase medication adherence, and these interventions could leverage widely available mobile technology tools such as mobile apps or text messaging, and existing routines.

## Introduction

### Background

Medication adherence is defined as taking medication exactly as prescribed [1]; this includes taking the proper dose at the right time. Medication adherence comprises three components: initiation, implementation, and persistence [2]. Medication nonadherence can occur at any of these three stages because of the failure to initiate a new prescription, implement it as prescribed, or persist with treatment [2]. Medication adherence is not a dichotomous variable (ie, adherence vs nonadherence) [2] but is more of a continuum (ie, variable levels of adherence). Lower adherence and variations in adherence can lead to loss of drug effectiveness, toxicity, and drug resistance [3]. Only approximately 50% of medications are taken as recommended in different patient populations [4-6], including children with chronic conditions [7,8]. The costs of low medication adherence are both personal and economic. In the United States, this has been shown as a cycle where poor medication adherence leads to poor patient outcomes [9-15] and increased service utilization and health care costs [9,10,12,16], all of which are passed down to the patient, further affecting adherence [17]. The Institute for Healthcare Informatics identified US $500 billion in savings across 186 countries with the responsible use of medication and noted that about 8% of the global total health expenditure could be avoided by improving adherence to medication [2].

Habit is the context-dependent automatic completion of a behavior [18]. Medication adherence would be an example of such a behavior where patients may take the same number of pills in the same room at the same time of day. Therefore, high habit strength is the result of recurring contextual cues [19]. As habit is automatic, it works independently of, and can even override, conscious desires when strong enough [19]. There are 2 types of medication nonadherence: intentional and unintentional [20]. Forgetfulness is the number one cause of unintentional nonadherence [21]. As habit is independent of conscious cognitive processes, having high habit strength protects against forgetfulness. Earlier research demonstrated a relationship between the strength of habit and medication adherence. Previous research examined habit’s direct effect on adherence and how habit interacts with more conscious factors to influence or overrule them [22]. However, the relationship between habit and adherence remains unclear.

Access to personal and mobile technology is ubiquitous [23-25], and there has been strong evidence to support the efficacy of digital or mobile health (mHealth) behavioral interventions, in particular text messaging and apps as tools to improve medication adherence [26-34]. These findings make mHealth interventions an appealing approach to optimize habit formation and medication adherence behavior in pediatric and adult patients with chronic health conditions [35]. However, the cost-effectiveness of these interventions remains unclear [36,37].

### Objective

This review aimed to systematically evaluate the most recent evidence for habit strength, medication adherence, and habit-based mHealth interventions across chronic medical conditions.

## Methods

### Study Design

The guidelines for the Preferred Reporting Items for Systematic Reviews and Meta-Analyses (PRISMA) were followed in the reporting of evidence across the studies reviewed herein [38]. The PRISMA checklist is included in Multimedia Appendix 1. To conduct this systematic review, a literature search was conducted on the PubMed database on June 25, 2019. Search terms were used in various combinations, including the following keywords: habit, habit strength, habit index, medication adherence, and medication compliance. For the first round of screening, 2 independent reviewers (RS and UB) conducted the keyword search and removed duplicates. Both the reviewers (RS and UB) then screened titles and abstracts independently for eligibility criteria and removed those that did not meet our inclusion criteria. Full texts were retrieved for the studies that were agreed on, and the 2 reviewers (RS and UB) completed full-text screening independently against our eligibility criteria. After conducting both screening steps, the results were compared, and any disagreements were settled by discussion with a third senior reviewer (SB).

### Eligibility Criteria

Eligible studies were original research studies in English and included validated quantitative measures of habit strength and medication adherence that have been used in earlier published studies. Studies examining all ages, conditions, and countries were included. The included studies needed to evaluate habits specifically in the context of taking medication. This excluded lifestyle habits and general habit formation such as smoking, diet, and exercise. Studies that looked at adding medication to preexisting habits were also excluded. We excluded studies that evaluated habit strength and medication adherence solely from qualitative interviews without any validated measures.

### Data Synthesis

A standardized form was used for data extraction. This form included the following categories: title, author, year of publication, country, number of participants, age, gender, study design, study approach, theoretical model, medical condition, habit strength instrument, adherence scale, measured habit strength, measured adherence rates, habit strength and medication adherence relationship (quantitative), main study conclusion, other study outcomes, and quality of the evidence. To assess the quality of the included studies, the Grades of Recommendation, Assessment, Development, and Evaluation (GRADE) criteria were used [39]. The GRADE approach evaluates a body of evidence by starting with a quality level based on the underlying methodology and then upgrading or downgrading the quality level based on various factors. Randomized trials or double-upgraded observational studies were rated as high. Downgraded randomized trials or upgraded observational studies were rated as moderate. Double-downgraded randomized trials or observational studies were rated as low. Triple-downgraded randomized trials and downgraded observational studies or case series/case reports were rated very low. Factors that downgrade the quality of evidence include limitations that suggest bias, indirectness of evidence, unexplained heterogeneity or inconsistency of results, imprecision of results, or a high probability of publication bias. Factors that improve the quality of evidence include a large magnitude of effect (ie, when all plausible confounding factors reduce a demonstrated effect or suggest a spurious effect when results show no effect) and dose-response gradient [38]. Data were analyzed and summarized qualitatively.

## Results

### Literature Search

**Figure 1:**
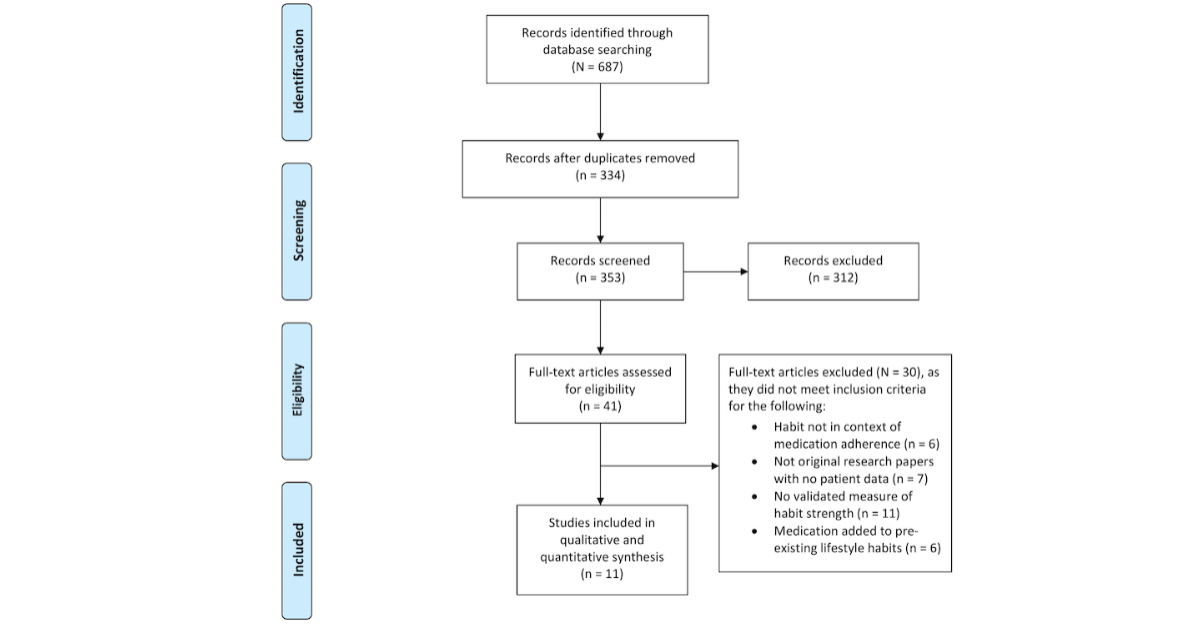
Flow of studies through the review according to Preferred Reporting Items for Systematic Reviews and Meta-Analyses (PRISMA) guidelines.

Our literature search identified 687 studies for screening (title and abstract). Of these, 41 full-text studies were reviewed, and 11 studies [9-12,20,22,40-44] met all inclusion criteria. This process is outlined in the PRISMA flow chart (Figure 1).

### Description of Included Studies

#### Study Characteristics

Table 1 summarizes the study characteristics. The research from the included studies was conducted across a range of countries, including 1 study from the Netherlands [22], 2 from Canada [10,20], 2 from Ireland [40,41], 3 from the United Kingdom [11,43], and 3 from the United States [9,42,44]. All the included studies were published over the past 9 years, with the oldest published in 2011 [22] and the most recent in 2019 [11]. All studies included studies on habit strength and medication adherence in a specific chronic disease population including asthma [22], type 2 diabetes [10,20,42], hypertension [9,40,44], cystic fibrosis [11,12], and psoriasis [43], except for 1 study that looked at a population taking oral contraceptives [41]. All studies included adult subjects, but the participants’ mean age ranged greatly from 22.41 to 69.86 years. The number of participants varied as well; the included studies ranged from 61 to 901 participants, with a mean of 331.82 participants and a median of 202 participants. The majority of the studies had a roughly equal ratio of male to female participants. One study observed a veteran population [44] with a mean age of 64.1 years, and only 14% women were included in the study [44]. Owing to the nature of the population, a study on oral contraceptives had a 100% female population [41]. Most studies (n=6) were cross-sectional [10,20,22,40,43], 4 were longitudinal studies [9,12,42,44], and 1 was a pilot randomized control trial [11]. In the included studies, medication adherence was measured using a combination of self-report questionnaires, remote monitoring using electronic pill bottles, and in-person interviews. The majority of studies (9/11, 82%) measured habit strength using the self-report habit index and self-report behavioral automaticity index [9-11,20,22,40-43].

**Table 1:** Summary of included studies that evaluated habit strength and medication adherence.

Source (country)	Health condition	Participants (N)	Age (years), mean (SD)	Sex (female), n (%)	Study design	Theoretical model	Study assessments	Quality of evidence^a^
Bolman et al [16] (Netherlands)	Asthma	139	31.5 (5.60)	99 (71)	Cross-sectional study	ASE^b^ model	Mail-out survey, questionnaire	Low
Burns et al [14] (Canada)	Type 2 diabetes	790	64.05 (8.20)	387 (49)	Cross-sectional study	—^c^	Telephone interview, questionnaire	Moderate
Durand et al [19] (Ireland)	Hypertension	204	69.86 (10.69)	86 (42)	Cross-sectional study	CS-SRM^d^	Questionnaire	Low
Guenette et al [8] (Canada)	Type 2 diabetes	901	62.70 (9.10)	369 (41)	Cross-sectional study	TPB^e^	Questionnaire	Very low
Hoo et al [10] (United Kingdom)	Cystic fibrosis	123	25.00^f^ (19-31)	52 (42)	Longitudinal study	Habit index measure	Electronic pill bottle	Very low
Hoo et al [9] (United Kingdom)	Cystic fibrosis	61	27.40 (21.70-37.10) - low adherence, 23.70 (18.40-32.00) - moderate adherence, and 26.10 (21.20-37.50) - high adherence^f^	28 (46)	Pilot randomized control trial	COM-B^g^ model	Questionnaire, electronic pill bottle	Very low
Murphy et al [20] (Ireland)	Oral contraceptive pill	245	22.41 (4.78)	245 (100)	Cross-sectional study	—	Questionnaire	Very low
Phillips et al [7] (United States)	Hypertension	71	67.9 (12.28)	45 (63)	Longitudinal study	CS-SRM	Interview, MEMS^h^	Low
Phillips et al [21] (United States)	Type 2 diabetes	103	56.96 (12.94)	64 (62)	Longitudinal study	CS-SRM	Interview, electronic pill bottle, Fitbit, survey	Very low
Thorneloe et al [22] (United Kingdom)	Psoriasis	811	48.10 (13.10)	349 (43)	Cross-sectional cohort study	CS-SRM	Questionnaire	Moderate
Voils et al [23] (United States)	Hypertension	202	64.10 (11.00)	28 (14)	Longitudinal study	—	Survey	Very low

^a^Quality of evidence assessed using the Grades of Recommendation, Assessment, Development, and Evaluation criteria.

^b^ASE: attitude, social influence, and self-efficacy model.

^c^Missing data were not reported in the included studies.

^d^CS-SRM: common sense model of self-regulation.

^e^TPB: theory of planned behavior.

^f^Median age (years) is reported when the mean age was not provided in the included studies. IQR in parenthesis.

^g^COM-B: capability, opportunity, motivation, and behavior.

^h^MEMS: medication event monitoring system.

### Description of Guiding Models

Different behavioral models exist to explain the process that occurs before a behavior takes place. In the context of this systematic review, the behavior being studied is medication adherence. Most studies (8/11, 73%) included theoretical models that comprised the guiding framework [9-12,22,40,42,43]. A variety of theoretical models were used by the included studies: the attitude, social influence, and self-efficacy model (ASE) [22]; the common sense model of self-regulation (CS-SRM) [9,40,42,43]; the theory of planned behavior (TPB) [10]; and the capability, opportunity, motivation, and behavior model (COM-B) [11].

The ASE is a behavioral explanatory model that takes a look at attitude, social influence, and self-efficacy as the predictors of intention and behavioral change [22]. Adapting the model to medication adherence, attitude is referred to as “the perceived pros and cons of taking medication,” social influence included “perceived norms and support of important others toward medication adherence and modeling which is the perceived behavior of others,” and self-efficacy was defined as “the person’s belief that they could adhere to medicine” [22]. Habit was observed as either a moderating or mediating factor in this model.

The CS-SRM proposes that an individual has a certain representation of an illness in their mind that guides how they respond to an illness threat [45]. The components of the illness representation are identity, causes, consequences, timeline, and controllability [40]. In the context of this systematic review, the response to illness threat would be medication adherence. According to the CS-SRM model, treatment-favorable beliefs lead to the initiation of behavior, and experiential feedback proves that those beliefs were correct (CS-SRM coherence), and the behavior was practiced until it became habit, leading to long-term medication adherence [9].

Fundamentally, the TPB states that attitudes, subjective norms, and perceived behavioral control create the intention to perform behaviors such as medication adherence [10]. When an individual is given sufficient control over their situation, intention will be turned into behavior when given the opportunity to do so [10]. The included study that used the TPB as a guiding model [10] examined habit as another factor that influences intention and the performance of medication adherence behavior.

The COM-B is a behavior system in which capability, opportunity, and motivation interact and lead to behavior [46]. The behavior itself, then, influences capability, opportunity, and motivation. Capability includes knowledge, cognitive ability, and physical skills to perform a behavior. Opportunity is defined as factors that lie outside the patient’s environment that make taking medications possible or prompt them to do so according to the cultural milieu, including the access to medications and medical care as well as the perceptions related to disease stigma. In other words, opportunity includes any factor that is not in the hands of the individual. Motivation energizes and directs behavior. Goals, conscious decision-making, habitual processes, emotional responding, and analytical decision-making are all components of motivation. In the context of this systematic review, habit is part of the automatic motivation that energizes and directs the behavior of medication adherence.

### Description of Habitat Strength Measures

Table 2 summarizes the habit strength and adherence measure as well as the main outcomes. The majority of studies (9/11, 82%) measured habit strength using the self-report habit index and self-report behavioral automaticity index [9-11,20,22,40-43]. The self-report behavioral automaticity index is a subset of the self-report habit index, and an example item from this index would be taking this medication is something I do automatically, which is rated on a scale of 1-5 from strongly disagree=1 to strongly agree=5 [21]. One study [9] modified this scale by adding 4 additional questions that evaluated the concept of habit strength more broadly and intuitively (ie, asking patients in different ways whether or not they have a habit of taking their medication vs asking them in different ways if they take their medication without conscious attention, without conscious awareness, etc). One of the studies [12] built and tested a new form of measuring habit strength, using the habit index scale as its guiding model. A total of 2 studies [12,44] used the multiplicative product of behavior frequency and context stability.

**Table 2:** Summary of habit strength, medication adherence measures, and outcomes in the included studies.

Source	Habit strength measure	Adherence scale and rates	Relationship between habit strength and adherence rates
Bolman et al [16]	SRHI^a^	MARS^b^	Correlation *r*=0.61; *P*<.001
Burns et al [14]	Self-report behavioral automaticity index	*Did you ever forget to take your medication?* on a 5-point scale	Depressive symptoms: beta=.08; *P*<.001; 95% CI 0.04 to 0.12 Diabetes distress: beta=.09; *P*<.001; 95% CI 0.04 to 0.12 Major depressive syndrome: beta=.07; *P*<.001; 95% CI 0.03 to 0.11
Durand et al [19]	Self-report behavioral automaticity index	Overall adherent range: 58.9%-79.7% MARS: 36.7% nonadherent MMAS^c^: 41.1% nonadherent Prescription refill: 79.7% adherent Urine assay Total nonadherence, 2.1% Partial nonadherence, 23.8%	MARS: correlation *r*=0.36^d^; *P*<.001 MMAS: correlation *r*=0.35^d^; *P*<.001 Prescription refill: correlation *r*=0.08 Urine assay: correlation *r*=−0.02 Adherence composite: correlation *r*=0.36; *P*<.001 Hierarchical regression analysis: beta=.44; *P*<.001; adjusted. R^2^=0.22, ΔR^2^=0.19; ; *P*<.001 Unintentional adherence: beta=−.45; t_203_=−7.04; *P*<.001 Intentional adherence: beta=−.22; t_203_=−3.08; *P*<.01
Guenette et al [8]	SRHI About 71% scoring *high* (at least 5/6) Mea	MMAS-8 modified French version 45% high adherence 40.7% medium adherence 14.3% low adherence	Adjusted OR^e^ 1.65; 95% CI 1.35 to 2.03; *P*<.001
Hoo^a^ et al [10]	Multiplicative product of behavior frequency and context stability	Electronic pill bottles 47.30% median adherence 4.9% low adherence 80.5% variable adherence 14.6% high adherence	Overall cohort: *R*=0.40; 95% CI 0.36 to 0.44; beta=.30; 95% CI −1.04 to 1.65 Adherence consistently low: *R*=0.24; 95% CI 0.04 to 0.44; beta=3.03; 95% CI −9.68 to 15.76 Variable adherence: *R*=0.45; 95% CI 0.41 to 0.49; beta=.08; 95% CI −1.44 to 1.60 Adherence consistently high: *R*=0.20; 95% CI 0.13 to 0.27; beta=.61; 95% CI −1.90 to 3.13
Hoo^b^ et al [9]	Self-report behavioral automaticity index	Chipped nebulizer 75.4% low adherence 13.1% medium adherence 11.5% high adherence	Median habit strength in different subgroups: Low adherence: 9.0, IQR 4.8-12.0 Moderate adherence: 14.5, IQR 11.3-18.3 High adherence: 18.0, IQR 14.0-20.0 All significantly correlated with adherence levels, *P*<.001
Murphy et al [20]	Self-report behavioral automaticity index Mean habit strength per number OCP^f^ missed per month	MARS Mean MARS score per number of OCP missed per month: Never: 5.85 Once: 7.49 Twice or more: 10.12	Correlation *r*=−0.24^g^; *P*<.001
Phillips et al [7]	Self-report habit index, with 4 additional questions	MARS, MMAS, MEMS^h^ Mean adherence MMAS=0.80 MEMS timing adherence=76% MEMS dosing adherence=96%	Bivariate relationship (correlations): MARS: 0.37 MMAS: 0.26 MEMS dose frequency: 0.42 MEMS dose timing: 0.49 Hierarchical regression analysis: MARS: ΔR^2^=0.11; *P*<.01 MMAS: ΔR^2^=0.06; *P*=.04 MEMS frequency: ΔR^2^=0.17; *P*<.001 MEMS timing: ΔR^2^=0.27; *P*<.001 Unintentional nonadherence: beta=−.32; t_66_=−2.55; *P*=.01 Intentional nonadherence: beta=−.23; t_66_=−1.82; *P*<.07
Phillips et al [21]	Self-report behavioral automaticity index Mean medication-taking habit strength 3.75	MARS and MEMS Mean adherence: MARS=4.66 Self-reported intentional nonadherence=1.24 Self-reported unintentional nonadherence=1.76 MEMS % days adherent=76.19 MEMS % doses on time=60.68	Bivariate correlations: MARS: 0.40, *P*<.001 Self-reported intentional nonadherence: −0.34; *P*<.001 Self-reported unintentional nonadherence: −0.41; *P*<.001 MEMS % days adherent: 0.37; *P*<.001 MEMS % doses on time: 0.40; *P*<.001 MARS (with control variables): beta=0.15; β=.32; *P*<.001 MEMS (with control variables): beta=8.57; β=.32; *P*<.01
Thorneloe et al [22]	Self-report habit index Mean 41.5 for self-administered systemic therapy	MARS Overall: 22.4%, nonadherent 12% intentional 10.9% unintentional Conventional: 29.2% overall 15.3% intentional 14.5% unintentional Biologic: 16.4% overall 9.1% intentional 7.7% unintentional	Multivariable regression model: 0.94 overall nonadherence: 95% CI 0.91 to 0.97 0.95 intentional nonadherence: 95% CI 0.92 to 0.98 0.92 unintentional nonadherence: 95% CI 0.89 to 0.96
Voils et al [23]	Product of frequency and mean of 5 situational consistency items	Patient rating and MMAS-8 60% nonadherent	Extent of nonadherence: correlation *r*=−0.39; *P*<.001

^a^SRHI: self-report habit index.

^b^MARS: medication adherence report scale.

^c^MMAS: Morisky medication adherence scale.

^d^Z-scores averaged. So greater MARS and MMAS scores represented greater nonadherence.

^e^OR: odds ratio.

^f^OCP: oral contraceptive pill.

^g^Negative because lower MARS score represents better adherence.

^h^MEMS: medication event monitoring system.

### Description of Adherence Measures

The most common measures of adherence were the medication adherence report scale [9,22,40-43] and the Morisky medication adherence scale [9,10,40,44]. Other measures of adherence included medication event monitoring systems [7,9,21], medication possession ratio based on prescription refill data [19], single item self-report [14], and urine drug monitoring [14].

### Study Methodological Quality

The quality of the included studies ranged from very low to moderate. Of the 11 included studies, 2 were of moderate quality [20,43], 3 were of low quality [9,22,40], and 6 were of very low quality [10-12,41,44]. Table 1 reports the quality of each included study.

### Description of Study Outcomes

#### Habit Strength, Medication Adherence, and Mobile Health Interventions

Table 3 summarizes the study outcomes related to habit strength and medication adherence. Most studies showed a positive correlation between habit strength and medication adherence behavior, suggesting stronger habit formation with higher medication adherence rates [9-12,22,40-44]. Furthermore, compared with factors such as pill burden [40], illness coherence [40], treatment-related beliefs [9,40], and experiences with treatment-related efficacy [9], habit strength was the strongest predictor of adherence. Habit strength had the strongest association with medication adherence and medication event monitoring system dose timing among all the other adherence measures in 1 study [9]. However, habit strength was found to be equally correlated to dose timing and days taken in a later study [42].

Habit strength was also found to mediate the effects of self-efficacy on adherence [22]. The effect of self-efficacy on adherence disappeared once habit strength was added to the hierarchical multiple regression analysis model, and this relationship was confirmed with bootstrapping analysis [22]. Social norms moderated the relationship between habit strength and medication adherence; in weak habit, a supportive norm of taking medicine was positively related to adherence, and supportive norms were only positively correlated with adherence when habit strength score was low [22].

Even after adjusting for covariates, such as age and disease duration, habit strength moderated the association between poor mental health symptoms and medication adherence [20]. Interaction between habit strength and depressive symptoms was also observed. When habit strength was weak or average, depressive symptoms were negatively associated with adherence [20]. However, if habit was strong, no association was observed [20]. This same interaction was observed between diabetes distress and habit strength as well as between major depressive syndrome and habit strength [20].

Habit strength was more strongly associated with unintentional nonadherence than intentional nonadherence in 2 studies [9,40] but was equally predictive in another study [42]. None of the included studies reported on using or proposing a habit-based mHealth behavioral intervention to promote medication adherence.

**Table 3:** Summary of the main study findings.

Source	Study outcomes
Bolman et al [16]	Higher habit strength is positively correlated with higher adherence. Habit mediates the relationship between self-efficacy and medication adherence. Social norms moderate the relationship between habit and adherence; in weak habit, a supportive norm of taking medicine was positively related to adherence, and in strong habit, supportive norm correlated with less adherence. Perceiving few negative consequences of taking medicine was associated with better adherence. Control variables of risk perception and asthma severity were positively correlated with adherence. Female gender was positively correlated with adherence. Control variable of internal locus of control negatively correlated with adherence. From the central concepts, perceiving more pros, social support, higher self-efficacy, and stronger habit was associated with more adherence. From the central concepts, habit strength and attitude pros had the strongest correlation with medication adherence. Social norm and modeling were not significantly associated with adherence. Social influence subscales were highly intercorrelated, as well as habit with risk perception, pros, social support, and self-efficacy. After hierarchical multiple regression, habit strength proved to be significantly related to adherence. Of the control variables, only severity remained significant; of the ASE^a^ concepts, only the cons remained significant.
Burns et al [14]	Interaction between habit strength and depressive symptoms was observed. If habit strength was weak or average, depressive symptoms were negatively associated with adherence. However, if habit was strong, no association was observed. Same significant interaction pattern was observed for diabetes distress and habit strength as well as major depressive syndrome and habit strength. Habit strength moderates the association between poor mental health symptoms and medication adherence. After adjusting for covariates, results remained significant.
Durand et al [19]	Medication-taking habit strength was the strongest predictor of adherence (compared with pill burden, illness coherence, and treatment-related beliefs). Habit strength explained 19% incremental variance in adherence beyond treatment-related beliefs. Habit strength was more strongly associated with unintentional nonadherence than intentional. Associations among adherence measures were weak to moderate, indicating that multiple measures are necessary to accurately assess adherence. Neither treatment-related beliefs nor CSM^b^ coherence predicted adherence, even for patients with weak habit strength. Pill burden was not associated with habit strength or adherence. There was no significant interaction between treatment-related beliefs, habit strength, and adherence.
Guenette et al [8]	Strong habit was significantly associated with adherence. Perceived behavioral control, older age, no perceived side effects, a longer period since T2D^c^ diagnosis, and a lower number of NAID^d^ daily doses were significantly associated with adherence. Sex, level of education, and income are not associated with adherence. Intention, insulin use, number and type of NIAD drugs prescribed, perceived cost of antidiabetes medications, and use of glucometer or weekly pill organizer were not associated with NIAD adherence. Depressed mood, anxiety, and mental health were not associated with adherence. Behavioral control was found to be significant, so the 26 underlying beliefs were analyzed, and 12 beliefs were found to be significant with adherence.
Hoo^a^ et al [10]	One unit increase in habit index was associated with a 0.3% increase in the subsequent week’s adherence after controlling for current adherence. Those with variable adherence displayed higher mean cross-correlation coefficients (0.45) compared with those with consistent adherence (0.20-0.40).
Hoo^b^ et al [9]	Higher adherers reported stronger habit compared with lower adherers. A 1-unit increase in habit strength was associated with a 31% increase in odds of being in the next higher adherence category. In a multiple ordinal regression model with both habit and concerns scores, only habit was associated with adherence. Higher adherers had lower prior year intravenous use, tended to have higher %FEV^e^ at baseline, and reported lower concerns.
Murphy et al [20]	Stronger habit strength was associated with better adherence. Those who never miss an OCP^f^ reported significantly higher habit strength than those who miss 2 or more per month. There was no difference between those who never miss an OCP and those who miss 1 OCP per month. Having a fixed time of day to take the OCP was associated with better habit strength and adherence. There is, however, no association between habit strength and taking OCP at different times of the day. Having a fixed place to store the OCP was associated with habit strength but not adherence.
Phillips et al [7]	Habit strength was the strongest predictor of medication adherence (compared with beliefs and experiences plus efficacy)—explains 6%-27% incremental variance in adherence to that explained by treatment-related beliefs. Habit strength was more strongly related to unintentional medication nonadherence than intentional nonadherence. Patients’ CS-SRM^g^ coherence was more strongly associated with intentional nonadherence than unintentional adherence. Patients’ treatment-related beliefs were not more strongly associated with intentional nonadherence than unintentional nonadherence. Habit strength had the strongest association with MEMS^h^ dose timing out of all the adherence measures. The interaction between treatment-related beliefs and habit was not significant for any of the adherence measures. Patients’ beliefs and experiences did not predict overall adherence, even for weaker adherence. Patient experience, however, did predict intentional nonadherence.
Phillips et al [21]	Habit strength consistently predicted incremental variance in measured outcomes, both self-reported and measured. Correlations, between habit strength and % of the doses taken on time vs between habit strength and % of the days when medications were taken, were not significantly different. Habit strength does not predict unintentional nonadherence better than intentional. Habit strength is not relatively more important for predicting medication adherence than physical activity.
Thorneloe et al [22]	Patients in the biological cohort were more likely to be male, have a younger age of onset of psoriasis, longer duration of disease, more likely to have a diagnosis of inflammatory arthritis, have lower quality of life scores at the start of therapy, have longer duration of systemic therapy, have stronger beliefs in the chronicity of their illness, stronger beliefs that systemic therapy is necessary, weaker concerns about therapy and medicine, greater coherence, and less symptoms of depression. Patients using self-administered systemic therapy had strong habit strength. Being on a conventional systemic therapy, having strong medication concerns, longer treatment duration, and younger age were factors associated with overall nonadherence. Being on a conventional therapy and strong medication concerns were also significant for intentional nonadherence. Being on a conventional systemic therapy, stronger perceptions of psoriasis being a chronic condition, younger age, and longer treatment duration were factors associated with unintentional nonadherence. Group 1 membership (strongest medication concerns) was associated with intentional nonadherence, and weaker medication-taking routine or habit strength was associated with unintentional nonadherence.
Voils et al [23]	Dual conceptualization (self-report with psychometric principles) of medication nonadherence has stronger validity and reliability than other forms that confound these 2 variables. Extent of adherence was highly correlated with self-efficacy, where lower adherence levels were associated with lower self-efficacy. In all, 3 items assessing the extent of nonadherence produced reliable scores. Correlations between the extent and harm subscales with habit strength were above 0.3. Correlations and comparison measures showed convergent and divergent validity. Predictive validity was evidenced by correlations between extent and BP^i^. Means of the reasons items were well below the scale midpoint, and several distributions were positively skewed and kurtosis. The Morisky scale did not measure a single underlying construct in this sample. The Morisky score was not correlated with BP.

^a^ASE: attitude, social influence, and self-efficacy model.

^b^CSM: common sense model.

^c^T2D: type 2 diabetes.

^d^NAID: noninsulin antidiabetic drugs.

^e^FEV: forced expiratory volume.

^f^OCP: oral contraceptive pill.

^g^CS-SRM: common sense model of self-regulation.

^h^MEMS: medication event monitoring system.

^i^BP: blood pressure.

#### Other Adherence-Related Outcomes

In addition to habit strength, other factors significantly associated with increased medication adherence included perceived negative consequences of taking the medication [10,11,22,43], perceived behavioral control [10], and older age [10,43]. Longer treatment duration was found to be significantly associated with adherence in 2 different studies. One study [10] concluded that longer treatment duration led to higher adherence because a longer period led to the development of habit. In contrast, another study [43] concluded that longer treatment duration led to higher overall and unintentional nonadherence. Strong concerns were also associated with intentional nonadherence [43]. Stronger perceptions of having a chronic condition and younger age were also associated with unintentional nonadherence [43]. Disease severity also affected adherence behavior. For those taking asthma medications, asthma severity was positively correlated with medication adherence [22]. Having a fixed time of the day for taking medications was associated with better habit strength and better adherence, but there was no association between habit strength and the time of the day medications were taken [41]. Having a fixed place to store the medication was associated with higher habit strength but not higher medication adherence [41].

In the included studies, factors found to not have a relationship with medication adherence were modeling [22], treatment-related beliefs [9,40], and pill burden [40]. The association of treatment-related beliefs was not stronger for intentional nonadherence than for unintentional nonadherence [9]. One study [40] found that common sense model–related coherence did not have a significant relationship with medication adherence; yet, another study [9] found that CS-SRM coherence was more strongly associated with intentional nonadherence than unintentional nonadherence. Demographics associated with adherence included sex [10,22], education level [10], and income [10]. Although social norms did moderate the relationship between habit strength and medication adherence, it did not have a significant relationship with medication adherence by itself [22]. One study [20] found that there was an interaction between habit strength and mental health symptoms; however, depressed mood, anxiety, and mental health by themselves were not associated with adherence [10]. Patient experiences did not predict overall nonadherence but did predict intentional nonadherence [9].

## Discussion

### Principal Findings

Our systematic review contributes to the literature on habit strength and medication adherence across chronic medication conditions. We found that habit strength was strongly correlated with medication adherence, with stronger habit being associated with higher medication adherence rates, regardless of the theoretical model and/or guiding framework. As the behavior becomes more automatic, there is less chance for an individual to forget to take their medicine. We also found that the effect of habit strength on adherence was also related to the individual’s self-efficacy, social norms, and mental health symptoms. This has been explained in earlier studies investigating the dual-process theories, where an individual’s behavior is a result of both deliberative/reflective processes and implicit/impulsive processes [47]. When conscious processes are strong, they might be able to overpower the automaticity of habits. It is also important to note that many of the social influence subscales, as well as habit, were highly intercorrelated in relation to risk perception, pros/cons of taking medication, social support, and self-efficacy [22].

In 1 study [40], it was determined that associations among adherence measures were weak to moderate. This indicates that multiple measures are necessary to accurately assess adherence, as was done in some of the included studies. In addition, self-report questionnaires run the risk of social desirability bias, so monitoring with electronic pill bottles or looking into prescription pharmacy refill records are other important and informative ways of measuring medication adherence.

Our findings in this review suggest that interventions to increase medication adherence could be more effective if they focused on developing a stronger habit among individuals. One way to build a stronger habit is by reminding the patient when it is time to take their medication [18], such as with pill bottle caps that light up when it is time to take the medication. Another approach to strengthen a patient’s habit of taking medication is to leverage technology-based interventions and remind the patient to take their medication by sending a text message or alert on the patient’s phone when it is time to take their dose. Interestingly, a recent review reported that a number of grants, funded by the US National Institutes of Health (2014-2018), were focused on developing and testing mHealth smartphone apps that were specifically designed to facilitate medication adherence behavior by reminding patients to take their scheduled medications, which could lead to the development of a habit [48]. Furthermore, a possible habit-based intervention to increase medication adherence is to incorporate the medication routine into existing lifestyle habits such as physical activity, mealtimes, or morning routines to develop a stronger habit, which should be explored further in future research. However, in this review, we focused our research question on the relationship between habit strength and adherence rates, solely in the context of medication-taking behavior. It is also important to note that most of the included studies in our review were of low quality, and the majority were observational studies, yet they are informative for the most recent evidence on habit strength and medication adherence.

### Strengths

Our review has some strengths. In all, two authors independently completed the search process at each stage of the systematic review process, following established methodology guidelines (PRISMA). Some of the included studies used multiple forms of measure for medication adherence, making adherence assessment more accurate. Despite having no eligibility restrictions on the year of publication, all the included studies were published between 2011 and 2019, indicating an increasing interest in the topic of habit strength and medication adherence.

### Limitations

It is important to note some of the limitations of this systematic review. Given that all the search results came from 1 database (ie, PubMed) during the literature search, it is possible that some relevant studies could have been missed during the process. However, most of the studies in other databases, such as PsycINFO, are also indexed in PubMed, and the chances of missing relevant studies are relatively less. Furthermore, many of the included studies used self-report questionnaires, an approach that has the inherent limitation of social desirability bias. In addition, most of the included studies were observational and cannot evaluate the direction or the cause-and-effect relationship between habit strength and medication adherence. Moreover, it is important to note that the range of countries represented in the included studies was limited and included only developed countries. This is important because lifestyle factors, prescribing practices, and social/cultural norms could be different in different countries, affecting both the development of habit strength and medication adherence behavior. Therefore, the inclusion of studies from only developed countries limits the generalizability of this systematic review. Moreover, examining the relationship between habit strength and medication adherence should extend beyond developed countries where all the included studies were conducted. Developing countries have different clinical and research settings, and gaining insight from studies conducted there would be essential for future wide dissemination and implementation efforts of adherence-promoting behavioral interventions. Finally, this systematic review looked primarily at chronic health conditions, and further research should investigate the connection between habit strength and adherence behavior in nonchronic conditions. In addition, future research should also assess the longitudinal relationship between habit strength and medication adherence to better understand their cause-effect association, given that most of the included studies were cross-sectional.

### Conclusions

In conclusion, stronger habit has been associated with higher medication adherence rates. This is consistent with published literature indicating that forgetfulness is the leading cause of unintentional medication nonadherence. All studies in the literature examined habit strength in the context of nonadherence. Future rigorous longitudinal studies are needed to examine the direction of the relationship between habit strength and medication adherence behavior.

## Appendix

Multimedia Appendix 1 Preferred Reporting Items for Systematic Reviews and Meta-Analyses checklist.

## Abbreviations

ASE: attitude, social influence, and self-efficacy model
COM-B: capability, opportunity, motivation, and behavior model
CS-SRM: common sense model of self-regulation
mHealth: mobile health
PRISMA: Preferred Reporting Items for Systematic Reviews and Meta-Analyses
TPB: theory of planned behavior
