# Correction: Habit Strength, Medication Adherence, and Habit-Based Mobile Health Interventions Across Chronic Medical Conditions: Systematic Review

**DOI:** 10.2196/63980

**Published:** 2024-07-08

**Authors:** Sherif M Badawy, Richa Shah, Usman Beg, Mallorie B Heneghan

**Affiliations:** 1 Department of Pediatrics Northwestern University Feinberg School of Medicine Chicago, IL United States; 2 Division of Hematology Oncology and Stem Cell Transplant Ann & Robert H. Lurie Children's Hospital of Chicago Chicago, IL United States; 3 Weinberg College of Arts and Sciences Northwestern University Evanston, IL United States; 4 Midwestern University Arizona College of Osteopathic Medicine Glendale, AZ United States

In “Habit Strength, Medication Adherence, and Habit-Based Mobile Health Interventions Across Chronic Medical Conditions: Systematic Review” (J Med Internet Res 2020;22(4):e17883) the authors made one addition.

In the “Acknowledgements” section, which previously appeared as:

This project was supported by the National Heart, Lung, and Blood Institute of the National Institutes of Health (K23HL150232, principal investigator: Badawy). The content is solely the responsibility of the authors and does not necessarily represent the official views of the National Institutes of Health.

The authors have added an additional paragraph preceding the original, as follows:

We wish to acknowledge MMAS Research LLC for the use of ©Morisky Widget MMAS-8 software. Morisky Widget MMAS-8 software is protected by U.S. copyright and trademark laws. Permission for use of Morisky Widget MMAS-8 software copyright registration number TX 8-816-517 is required and was obtained for this research. A license agreement is available from MMAS Research LLC, 1501 Spring Hill Red, Petaluma CA, 94952 USA;  strubow@morisky.org. We would like to express our gratitude to Steve Trubow for the generous training and the timely license provision.

The correction will appear in the online version of the paper on the JMIR Publications website on July 8, 2024, together with the publication of this correction notice. Because this was made after submission to PubMed, PubMed Central, and other full-text repositories, the corrected article has also been resubmitted to those repositories.

